# A robust method for diagnosis of morphological arrhythmias based on Hermitian model of higher-order statistics

**DOI:** 10.1186/1475-925X-10-22

**Published:** 2011-03-28

**Authors:** Saeed Karimifard, Alireza Ahmadian

**Affiliations:** 1 Department of Biomedical Systems & Medical Physics, Tehran University of Medical Sciences, TUMS, Tehran, Iran; 2 Research Center for Science and Technology in Medicine, RCSTIM, Tehran, Iran

## Abstract

**Background:**

Electrocardiography (ECG) signal is a primary criterion for medical practitioners to diagnose heart diseases. The development of a reliable, accurate, non-invasive and robust method for arrhythmia detection could assists cardiologists in the study of patients with heart diseases. This paper provides a method for morphological heart arrhythmia detection which might have different shapes in one category and also different morphologies in relation to the patients. The distinctive property of this method in addition to accuracy is the robustness of that, in presence of Gaussian noise, time and amplitude shift.

**Methods:**

In this work 2^nd^, 3^rd ^and 4^th ^order cumulants of the ECG beat are calculated and modeled by linear combinations of Hermitian basis functions. Then, the parameters of each cumulant model are used as feature vectors to classify five different ECG beats namely as Normal, PVC, APC, RBBB and LBBB using 1-Nearest Neighborhood (1-NN) classifier. Finally, after classifying each model, a final decision making rule apply to these specified classes and the type of ECG beat is defined.

**Results:**

The experiment was applied for a set of ECG beats consist of 9367 samples in 5 different categories from MIT/BIH heart arrhythmia database. The specificity of 99.67% and the sensitivity of 98.66% in arrhythmia detection are achieved which indicates the power of the algorithm. Also, the accuracy of the system remained almost intact in the presence of Gaussian noise, time shift and amplitude shift of ECG signals.

**Conclusions:**

This paper presents a novel and robust methodology in morphological heart arrhythmia detection. The methodology based on the Hermite model of the Higher-Order Statistics (HOS). The ability of HOS in suppressing morphological variations of different class-specific arrhythmias and also reducing the effects of Gaussian noise, made HOS, suitable for detection morphological heart arrhythmias. The proposed method exploits these properties in conjunction with Hermitian model to perform an efficient and reliable classification approach to detect five morphological heart arrhythmias. And the time consumption of this method for each beat is less than the period of a normal beat.

## Background

**Figure 1:**
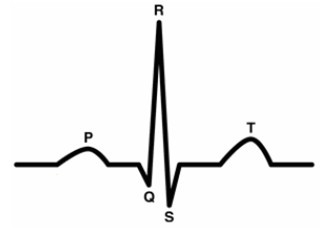
**A normal ECG beat**.

Electrocardiogram (ECG) signals (Figure 1), reflect electrical activities of heart and can be utilized in diagnosing arrhythmias. The development of an accurate, non-invasive and robust method for arrhythmia detection could assists cardiologists in the study of patients with heart diseases. Heart arrhythmias could be divided into two main groups of non-morphological and morphological arrhythmias. The amplitudes and positions of the main peaks of the signal such as QRS complexes are the main features for diagnosing non-morphological arrhythmias; sinus tachycardia and bradycardia are non-morphological arrhythmias. However, the shape and pattern of the signal may vary according to the type of heart disorder in morphological arrhythmias; premature ventricular contraction and bundle blocks arrhythmias belong to this group. The main signal peaks may be lost or undesired peaks may be appeared in morphological arrhythmias. In the case of such arrhythmias the morphology of ECG beats may also vary from beat to beat and patient to patient and can be affected by parameters such as age, sex and mental situation of the patients.

ECG signal morphology detection studies are commonly based on the signal modeling approaches. The Markov [1], Hermitian [2]- [3]- [4], Mathematical [5], Autoregressive (AR) [6] and Dynamic [7] models are the most popular methodologies for ECG modeling and heart arrhythmia detection. Although model-based methods are found successful in modeling of the arrhythmias, but the parameters of the model should be enough sensitive to be able to trace the variations occurred in the shape of the signal. This variation can potentially lead to make wrong decisions in the classification step as it will be shown in this paper. Higher-order statistics based methodologies are shown effective in suppressing such signal variations, in particular in the presence of noise, [8]. Using whole samples of the cumulants result large feature vectors which make the classification step more complex and time consuming [9]. In this study, we have proposed a new approach to reduce the dimension of the feature vectors by using the parameters of the modeling of cumulants based on the Hermitian basis functions. The effectiveness of Hermitian basis functions in modeling ECG signals has already been shown in [2]- [3]- [4]- [10]. Since the cumulants of the ECG signals have the same morphology as the original signal, it is expected that the Hermitian basis functions could be effective for cumulants modeling. In this paper properties of HOS in combination with Hermitian model parameters are used to detect morphological heart arrhythmias in a more stable and accurate manner.

Five different types of ECG beats are investigated in this study: The Normal sinus rhythm (N) and morphological arrhythmias including Atrial premature contraction (APC), Premature Ventricular Contraction (PVC), Right bundle branch block (RBBB) and Left bundle branch block (LBBB) [11].

We have utilized the 2^nd^, 3^rd ^and 4^th ^order cumulants to detect different ECG beats. To shortened the feature vectors, Hermitian basis function were used to model the cumulants, and the parameters of the model used as the feature vector. Three separate 1-NN classifiers are used to classify feature vector of each cumulants. Finally, by proposing a proper decision making rule the arrhythmias is classified according to the results of 1-NN classifiers.

The idea of combining the Hermite model and HOS in the approximation of ECG signals following by applying a suitable decision rule, results in an accurate method of arrhythmia detection and classification. The method is found reasonably robust to time/amplitude shifts and the additive Gaussian noise. Two subjective parameters, sensitivity and specificity are used to measure the efficiency of the method.

## Materials

In this section we first begin by describing basic concepts and the main properties of Higher-Order Statistics. Then the definition of Hermitian basis functions and the mathematical approach that they are used in signal modeling are explained. In the final section, the principles of 1-NN classifier which is used as the preliminary step for the final decision rule are described.

### Higher-Order Statistics

The second generating function of a random variable *x *can be defined as follow [12]:(1)

Cumulants are the coefficients of the Taylor series of the second generating function, *c_k _*defined as:(2)

The *n^th ^*cumulants of the signal are calculated as:(3)

where *τ_i_*'*s *are the time differences and  represents the *n^th ^*moments of the random variable *x*, that is obtained from the following equation:(4)

and  is the *n^th ^*order moment function of an equivalent Gaussian signal that has the same mean and autocorrelation sequence as *X*(*k*) For a zero mean sequence, 2^nd^, 3^rd ^and 4^th ^cumulants are calculated respectively as:(5)(6)(7)

In the above equation, *x_i _*denotes the *i^th ^*sample of the random variable *x*. The 2^nd^, 3^rd ^and 4^th ^orders of cumulants represent the variance, skewness and kurtosis of a signal [12] and were used here to generate the feature vectors. The three cumulants are suitable for analyzing non-Gaussian signals such as the ECG signals. The attractive properties of HOS exploited in this work are listed below [13]:

• Cumulants are additive to in their arguments.(8)

• Cumulants are blind to additive constants(9)

• Cumulants of a sum of statistically independent quantities equal the sum of the cumulants of the individual quantities.(10)

### Modeling based on Hermitian Basis Functions

Hermite polynomials, *H_n_*(*t*) are defined in the range of (-∞, +∞) and can be calculated by the following recursive formula and the initial values as:(11)

Hermite polynomials are not orthogonal in general, but can be modified to be orthogonal by multiplying them with an exponential factor as below [14]:(12)

where *ϕ_n_*(*t*,*σ*) is the Hermitian basis function,  are Hermitian polynomials; *σ *is the width parameter which controls the dilation and contraction of the basis functions; *n *is the order of the Hermite polynomial and the basis function.

**Figure 2:**
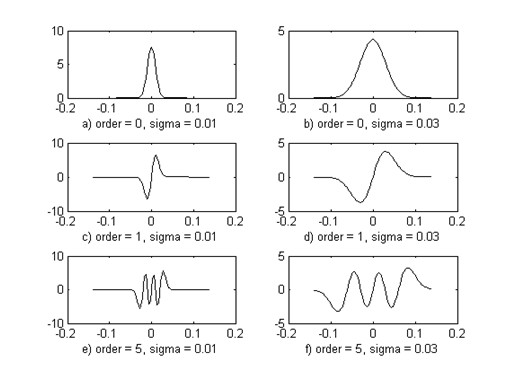
**Effect of order and width parameter on the Hermitian basis functions**.

Figure 2 shows the effects of order and width parameters on the shape of the Hermite basis functions. As it is seen in this figure, by increasing the order of the model, n, number of oscillation of the function increased. The number of functions peaks equal to (n + 1). Another parameter which influences the shape of the function is width parameter, *σ*. By increasing this parameter the width of the function is increased, as shown in Figure 2. Since Hermitian basis functions are orthogonal functions, any arbitrary signal can be approximated using a linear combination of these functions. Due to the similarity between low order Hermitian basis functions and the ECG beats, it is expected to approximate ECG beats by using fewer number of Hermitian basis functions. Since the cumulants of an ECG beat, approximately, have the same morphology as the original signal, it is expected that these functions are also suitable for modeling the cumulants as the signal itself.

### The Classifier

After calculating the parameters of the model for each cumulants, three feature vectors were constructed from every ECG beat. There have already been many methods for classifying ECG beats like support vector machine [4], multi-resolution analysis and neural networks [15], adaptive method [16] and neuro-fuzzy [17]. In this study, the 1-NN classifier is used because of its simplicity and fastness which was proved to have acceptable performance in arrhythmia classification, as shown in previous study [10]. Irrespective of the class labels, the general k-NN classifier categorizes different arrhythmias by identifying *k *nearest neighbors out of *N *training vectors. It later identifies the number of vectors that belongs to an arrhythmia class among these *k *samples and assigns the input sample to a class with the maximum number of samples. The k-NN classifier separates different types of ECG beats according to the following steps [18]:

• Out of N training vectors, identifying k nearest neighbors, irrespective of class label.

• Among these k samples, identifying the number of vectors, *k_i _*that belongs to an arrhythmia class (*ω_i_*).

• Assigning the input sample *x *o an arrhythmia class with the maximum number, *k_i _*of samples.

The nearest neighbor can be calculated using various distance measures such as Euclidean distance, sum of absolute differences and correlation measures; the Euclidean distance was used as the distance measure in this study.

### MIT/BIH Database

In this study, MIT/BIH heart arrhythmia database is used as the reference for five different types of ECG beats; normal beat and four different arrhythmia beats as APC, PVC, RBBB and LBBB. It is tried to recruit all beats of the arrhythmic signals in this database which results the number indicated in Table 1, and 2000 beats are selected randomly from normal beats. Among these amounts of beats, 60 percent are selected randomly as training beats, and remaining 40 percent are used as test data which is shown in Table 1. The ECG signals were obtained from MIT/BIH heart arrhythmia database available at the sample rate of 360Hz [11].

**Table 1:** Number of train and test beats of each type of ECG beats

Beat Type	Number of the Beats	Number of Training Beats	Number of Test Beats
**N**	2000	1200	800
**PVC**	2938	1763	1175
**APC**	722	433	289
**RBBB**	2251	1351	900
**LBBB**	1456	874	582

## Methods

**Figure 3:**
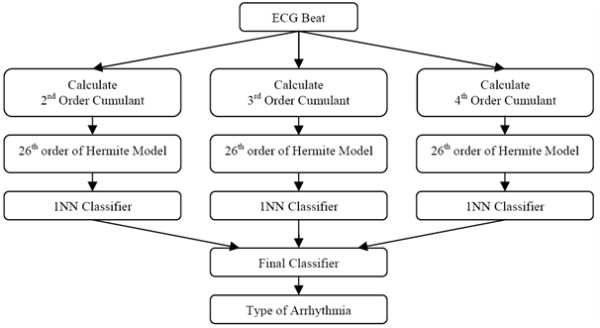
**Block diagram of the suggested methodology**.

The block diagram of the main steps of the proposed arrhythmia detection procedure is shown in Figure 3. As it is shown in this figure, at first 2^nd^, 3^rd ^and 4^th ^order cumulants are calculated and then modeled by Hermitian basis functions. The parameters of the model of each cumulants are classified using 1-NN classifier. The results of each three classifier are being input for a final decision maker which is described in the previous section. The details of the proposed steps in Figure 3are described below.

### Properties of cumulants

**Figure 4:**
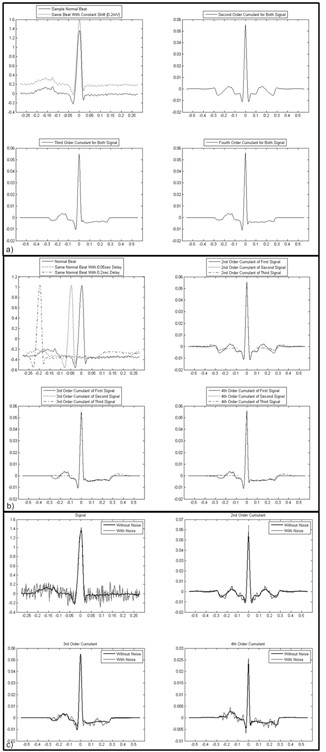
**A sample ECG beat and its cumulants**. a) The shift effect across the amplitude axis. b) The shift across time axis. c) The additive Gaussian noise effect.

In order to show the proper choice of cumulants for ECG signal processing, different experiments are performed. We have investigated the property of cumulants in eliminating the time/amplitude shifts and the additive Gaussian noise. Figure 4 shows the calculated cumulants versus the number of samples, normalized to the sampling rate. Figure 4a shows that the cumulants are able to eliminate the shift effect across the amplitude axis. Figure 4b shows that the cumulants could reduce the shift effect across the time axis. Figure 4c shows the Gaussian noise reduction effect.

**Figure 5:**
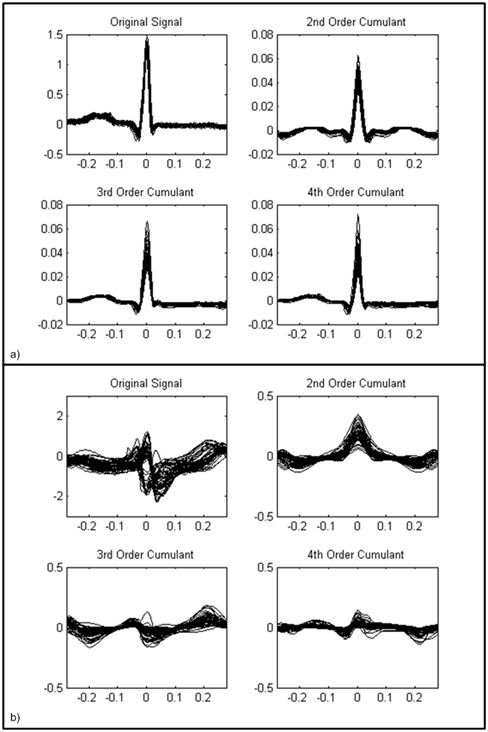
**Cumulants of normal ECG beats and PVC arrhythmia beats.** a) Samples of Normal ECG beats and their cumulants. b)Samples of PVC beats and their cumulants.

The other interesting property of cumulants is their ability in suppressing morphological variations of class-specific ECG arrhythmias. It can be seen for a series of normal ECG beats in Figure 5a, and PVC arrhythmic beats in Figure 5b, that the variation range at the R peak is much larger than the corresponding changes in their cumulants. For example, the amplitudes of Normal beats shown in Figure 5a, vary from 1.17 to 1.462 about the middle sample which is an interval length of 0.292. While this length is reduced to 0.0289, 0.0403 and 0.0493 for their corresponding 2^nd^, 3^rd ^and 4^th ^order cumulants, respectively. We observed that the relative variability of the amplitude of a signal's cumulants is less than the signal itself. This effect was also observed in other types of ECG beats and is in accordance with a previous work [4].

### Modeling the Cumulants

An accurate arrhythmia detection methodology should consider the differences in the morphologies of class-specific ECG beats and cumulants are shown to be an effective tool in this field. Implementing all samples of Cumulants constructs lengthy feature vectors. We have used a model of the Cumulants utilizing the Hermitian basis functions which prepares smaller feature vectors while maintaining the shape of the Cumulants. Each cumulants of the original ECG signal, *x*(*t*) could be approximated by linear combination of Hermite basis functions as the original signal itself:(13)

where *a_n_*'s are the coefficients of this linear combination; ϕ*_n_*(*t*,*σ*) are the Hermitian basis functions; *N *is the order of the model; *σ *is the width parameter. The two major parameters, *N *and *σ *should be optimized to achieve an acceptable approximation error in this model.

**Figure 6:**
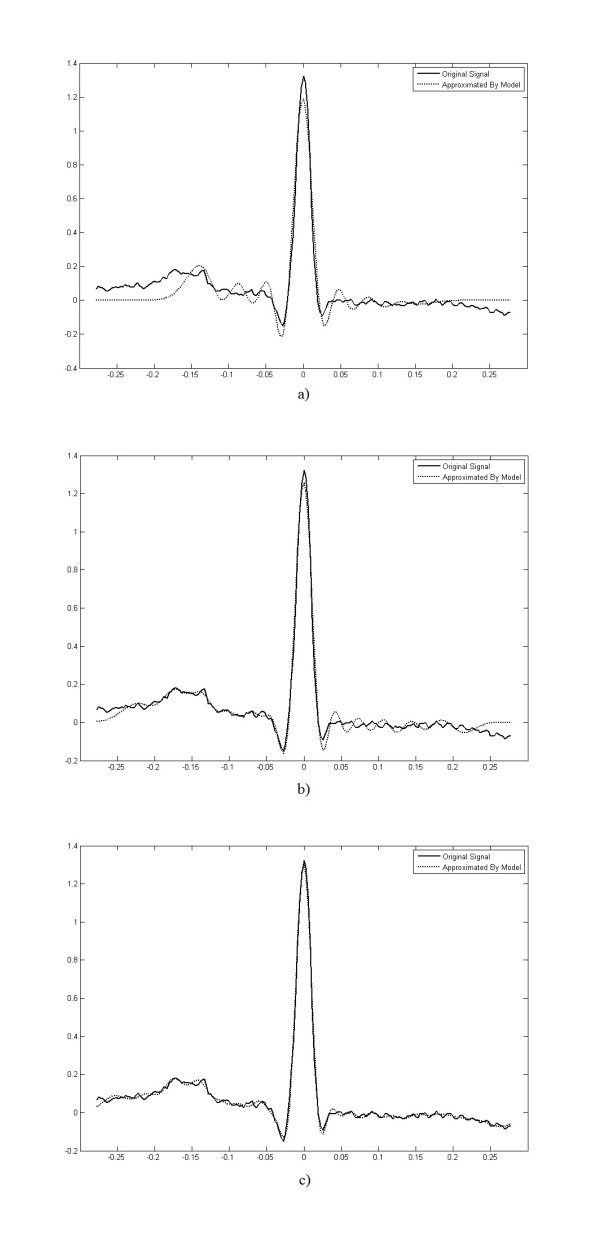
**Hermitian model of ECG beat at different orders.** a) Hermitian model of an ECG beat using order 15. b) Hermitian model of an ECG beat using order 25. c) Hermitian model of an ECG beat using order 35.

Figure 6 shows that high rate of change in the time domain can be observed if high orders of the Hermite basis functions are selected. Correspondingly, small changes of ECG beats can be detected. Increasing *N *and employing more Hermitian basis functions in the equation (13) increases the precision of the model and reduces the normalized approximation error, *E *defined as:(14)

**Figure 7:**
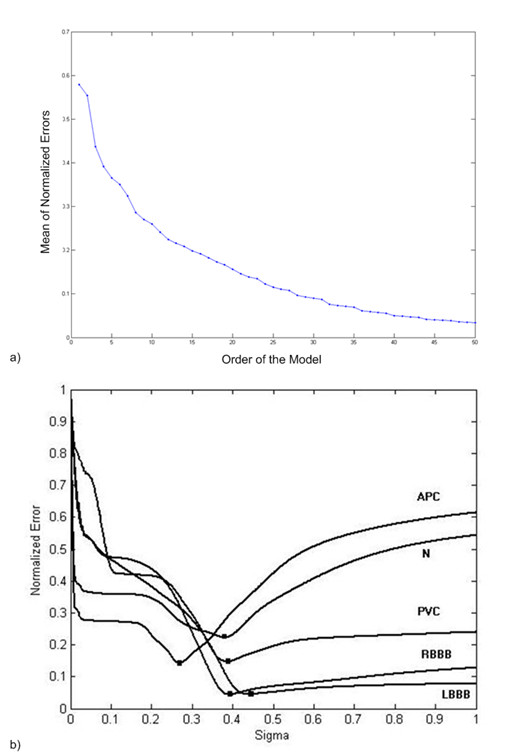
**Effect of model parameters on the approximation error.** a) Normalized error versus order of the model. b) Effect of width parameter on the approximation error.

The minimum acceptable approximation error can specify the order of the model. Figure 7a shows that the normalized approximation error exponentially decreases by increasing the order of the model.

Although increasing the order of the model decreases the approximation error but this process will add extra coefficients to the feature vector. These extra coefficients may decrease the weight of the significant coefficients and consequently reduce the accuracy of the classification. The effect of increasing the model order on the accuracy of the arrhythmia classification is shown in Table 2. It is observed that N = 26 a turning point for the sensitivity and specificity of classification. By increasing the order of the model the approximation of the signal become more accurate, but classification results are become worse. The reason of this event is that some coefficients which are only used for tracking small oscillation of the signal are added to the end of the feature vector by the same weight as the beginning coefficients, thus the results of classification using 1-NN classifier disturbed.

**Table 2:** Results of the classification of 5 different ECG beat according to different orders of the model

Order of the Model	Sensitivity (%)	Specificity (%)
**5**	93.89	98.57
**10**	96.82	99.26
**15**	97.84	99.49
**20**	98.45	99.64
**21**	98.45	99.64
**22**	98.72	99.71
**23**	98.64	99.68
**24**	98.82	99.73
**25**	98.45	99.64
**26**	98.88	99.74
**27**	98.42	99.63
**28**	98.48	99.64
**29**	98.42	99.63
**30**	98.58	99.67
**35**	98.69	99.69
**40**	98.21	99.58
**45**	97.76	99.47
**50**	96.98	99.29

The total computational time is important while modeling and classification routines should be completed in less than an ECG beat interval. We found that order 26 was suitable for our application. Accordingly, all the three cumulants are modeled in about 0.51 second. While the classification time is around 0.1 second, the total processing time takes about 0.6 second to complete which is less than the duration of a normal ECG beat. These tests are performed with the following hardware specifications:

• CPU: AMD 2200+

• RAM: 512MB

We observed that by optimizing the width parameter, the approximation error becomes considerably small. Figure 7b shows the effect of width parameter on the approximation error. We have utilized the genetic algorithm to optimize the width parameter. The genetic algorithm is a subset of evolutionary algorithms that models biological processes in order to optimize an involving cost function [19]. We have defined the approximation error as the cost function and set the mutation probability to 0.9. Setting mutation probability to 0.9 is because of that, if this number is bigger than 0.9, the algorithms may reach the optimum answer too late and if this number is set to a number less than 0.9, the algorithm may remain in a local minimum and could not find the real answer. In this study, every generation of the genetic algorithm consists of 10 width parameters. In each generation, the most two fit elements are chosen as the parents of the next generation. Implementing the following crossover functions,(15)

eight new offspring are created and by including two parents, ten new width parameters are ready for the next generation.

In order to model the ECG beats, according to the MIT/BIH database, the R peak is selected as the matching point of the model and 100 samples in either side of R peak are considered for processing. Referring to the sample frequency of the MIT/BIH database, these numbers of samples cover the main part of the ECG beats [9]. Accordingly, equation (13) consists of 201 linear equations and 26 undefined parameters. We may rewrite equation (13) in a matrix form as:(16)

where Φ is not a square matrix and is noninvertible. We have utilized the pseudo-inverse methodology which is itself based on least square error minimization method to obtain *a_n _*[14]. The pseudo-inverse methodology tries to find the inverse matrix Φ^† ^in order to calculate the coefficient matrix *A *from *X*.

### Classification and decision making

We performed some experiments to find out the effect of different cumulants in the preciseness of arrhythmia detection. The results of these experiments are shown in Table 3. According to these experiments, in 94.85% of cases the outputs of 1-NN classifiers for all three cumulants appeared the same which is shown in the "Occurrence Percentage" column in Table 3, and in 99.04% of cases the correct arrhythmias were detected. In some cases, the results of 2^nd ^and 3^rd ^order cumulants and 3^rd ^and 4^th ^order cumulants become the same by which the correct arrhythmias were found in 71.43% and 76.92% cases, respectively,. In 0.77% of cases, the results of 2^nd ^and 4^th ^order cumulants are the same but only less than half of the cases (48.28%) are detected correctly. The percent of cases in which the results of all three classifier become different were 0.64. Among them the results of third order cumulant are found more precise than the others.

**Table 3:** The results of ECG arrhythmia classification by using different sets of cumulants

Order of the cumulants with the same results	Occurrence (%)	True Classification (%)	False Classification (%)
**2,3 and 4**	94.85	99.04	0.96
**2 and 3**	1.31	71.43	28.57
**2 and 4**	0.77	48.28	51.72
**3 and 4**	2.43	76.92	23.08
**Only 2**	0.64	4.17	95.83
**Only 3**	0.64	54.17	45.83
**Only 4**	0.64	20.83	79.17

However, in conclusion from these experiments it can be said that among three cumulants, the results of the third cumulant are more precise. But using the other two cumulants makes the results more accurate. By these explanations two situations may occur after classifying each three feature vectors obtained from cumulants of an ECG beat, separately:

• First, at least two of the three different feature vectors are classified in the same class. In this case, the most repeated class will be chosen as the final output of the classifier.

• Second, each feature vector is classified in different classes, which means that three different types of ECG beats are nominated for the final classification by the same weight. In this situation, there must be a firm rule to select a definite class for such cases. For this purpose, some experiments were performed to find which cumulants has more reliable result, statistically. As described before, Table 3 shows that the 3^rd ^order cumulant achieved the highest accuracy. In these experiments, 0.64% of cases have three different results, and according to the information of Table 3, the third cumulants has the highest accuracy among the other cumulants (54.17% correct result). Thus, in the situation that three different classes are nominated for the final type of the ECG beat, we rely on the third cumulant result which leads us to a more accurate beat classification.

## Results

In our experiment, the results were based on using 9367 different ECG beats belonging to five different aforementioned types of ECG beats. Among these beats, 5621 beats were used as training sets and 3746 beats were used as test sets. For every ECG beat type, the total numbers of training and test beats are shown in Table 1. The statistical measures, Sensitivity (Se) and Specificity (Sp) have been selected to evaluate the accuracy of the model. By definition: , and ; where the True Positive (TP), False Negative (FN), False Positive (FP), and True Negative (TN) stand for the number of truly detected events, erroneously rejected events, erroneously detected events, and correctly rejected events, respectively.

**Figure 8:**
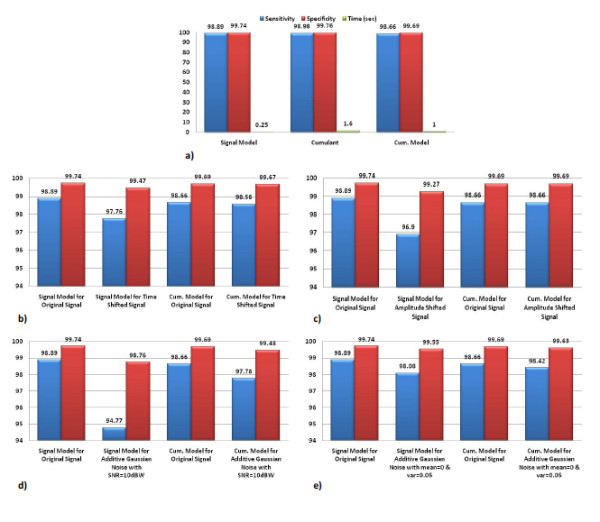
**Comparison evaluation of three methods and their robustness against time/amplitude shift and additive noise.** a) Comparison between the results of the combined feature vector with Hermite model and cumulants. b) Comparison between the effects of time shift on the signal model and cumulant model of the signal. c) Comparison between the effects of white Gaussian noise with SNR = 10dBW on the signal model and cumulant model of the signal. d) Comparison between the effects of amplitude shift on the signal model and cumulant model of the signal. e) Comparison between the effects of white Gaussian noise with zero mean and variance of 0.5 on the signal model and cumulant model of the signal.

Table 4, Table 5 and Table 6 presents the arrhythmia classification results utilizing the three methods: the cumulant model parameters (suggested method), signal model parameters and total cumulants' samples, respectively. Figure 8a compares the combined feature vector method with the method utilizing the parameters of Hermite model and the cumulants method. The methodology utilizing the parameters of the model has faster classification routine than the one utilizing only the cumulants; the model reduces the length of the feature vector while preserving the accuracy. Although utilizing the model of the signal achieved slightly better results than suggested methodology, the result is very sensitive to small variations of the signal and noise. We have also compared the stability and robustness of the suggested methodology to the Hermit model to verify our claim.

**Table 4:** Results of arrhythmia classification using cumulant model parameters (method 1).

Beat Type	Total Number	TP	TN	FP	FN	Sensitivity (%)	Specificity (%)
**N**	800	800	2941	5	0	100.00	99.83
**PVC**	289	276	3445	12	13	95.50	99.65
**APC**	1175	1155	2556	15	20	98.30	99.42
**RBBB**	900	895	2839	7	5	99.44	99.75
**LBBB**	582	570	3153	11	12	97.94	99.65

				**Average**		**98.66**	**99.67**

**Table 5:** Results of arrhythmia classification using signal model parameters (method 2).

Beat Type	Total Number	TP	TN	FP	FN	Sensitivity (%)	Specificity (%)
**N**	800	799	2936	10	1	99.87	99.66
**PVC**	289	272	3444	13	17	94.12	99.62
**APC**	1175	1161	2562	9	14	98.81	99.65
**RBBB**	900	896	2838	8	4	99.55	99.72
**LBBB**	582	576	3162	2	6	98.97	99.94

				**Average**		**98.89**	**99.72**

**Table 6:** Results of arrhythmia classification using the suggested method (Method 3).

Beat Type	Total Number	TP	TN	FP	FN	Sensitivity (%)	Specificity (%)
**N**	800	800	2943	3	0	100.00	99.90
**PVC**	289	272	3450	7	17	94.12	99.80
**APC**	1175	1166	2559	12	9	99.23	99.53
**RBBB**	900	896	2835	11	4	99.55	99.61
**LBBB**	582	574	3159	5	8	98.62	99.84

				**Average**		**98.98**	**99.75**

Although the results of the signal model and Cumulants are better than the suggested model by about 0.2 to 0.3 percent in sensitivity and about 0.1 percent in specificity, but it seems reasonable to ignore this amount of decrease against what we gain in the stability of the method as it is shown in Figure 8.

### Time-shift Effect

The ECG signal was shifted by one sample which is about 2.8 milliseconds and the classification results of the suggested methodology and Hermite model of the signal are shown in Figure 8b. The suggested methodology achieved higher accuracy compared to the signal model.

### Amplitude-shift Effect

The ECG beats are shifted up by 0.1 millivolts and the classification results of the both methodologies are shown in Figure 8c. While the amplitude shift produced a great effect on the results of the signal model, it is eliminated utilizing the suggested methodology.

### White Gaussian Noise Effect

One type of noise which presents in ECG signals is Electromyogram-like noise. This type of noise could be modeled by random white Gaussian noise [20]. This noise is found suitable to model artifacts such as motion artifacts, environmental noises or bioelectrical artifacts like Electrogastrogram (EGG). Thus in this experiment we examine the effect of white Gaussian noise on the suggested methodology and the signal model method. First, white Gaussian noise with *SNR *= 10*dBW *was added to the ECG signal. The noise considerably affected the signal model compared to the suggested methodology as is shown in Figure 8d. Second, white Gaussian noise of zero mean and variance of 0.05 and independent from the original signal is added. The signal model remarkably affected as is shown in Figure 8e.

## Discussions

It is important to note that we observed during our experiments that increasing the order of the model greater than 26, led to decrease the accuracy of the classifier output as described in the "method" section. This order of the model could be used as an optimum value comprising the computational cost and accuracy of the method.

Sixteen types of ECG beats and 109627 beats were considered in [2] which 74854 beats of them were normal beats. The sensitivity and specificity of beat type detection in [2] were good but not as good as the suggested method in this paper. However, because of the greater amount of beats and their types, a tolerable comparison is not possible. There is no data about the time consumption of the algorithm [2] and also robustness of the system in existence of time or amplitude shift and additive Gaussian noise, thus a detail comparison could not be accomplished.

Seven types of ECG beat and 7279 beats were used in [3]. The feature vector of [3] was the coefficients of the linear combination of the first 15 Hermitian basis functions as a part of current study and [10], using different classifier. It seems that the simpler and faster 1-NN classifier has approximately the same result as Neuro-Fuzzy network in beat type detection. On the other hand, the effect of time and amplitude shift and also additive Gaussian noise are considered in this study which was not in [3].

According to the number of beat types in [4] which was 13 types, cause of the more error detection in [4] rather than current study is explainable. The difference between these two methods is the difference in using HOS and the classifier. In [4] a few samples of 2^nd^, 3^rd ^and 4^th ^order cumulants are used in feature vector which is in current study the model of the cumulants are used. It is expected that having the model of the cumulants could results more accurate and comprehensive feature vector than a few samples of cumulants. It is also expected that having the model of cumulants which means the total cumulants of each beat, could depress the effect of time and amplitude shift and also additive Gaussian noise more accurately. As mentioned before, according to the comparison between the results of [3], [4] and [10], it seems that the more complicated classifier do not have better results in this case.

In comparison with [8], it could be said that the number of beats in [8] whish was using 2^nd^, 3^rd ^and 4^th ^order cumulants as the feature vector and different methods of fuzzy hybrid neural networks as the classifier, were less than the current study but types of the beats were not. The recognition rate current study is more than [8], but because of the differences between the sample beats and types, a correct comparison could not be done. It seems that the method used in [8] is fast, but there is no data about the stability of the method in existence of time and amplitude shift and additive Gaussian noise.

The suggested methodology also achieved higher accuracy compared to the autoregressive method [6]. In general, AR methods fail to model in noisy situations; we expect the suggested methodology to outperform the AR model in the presence of the noise.

## Conclusions

A novel and robust methodology is developed utilizing the cumulants and the Hermitian basis functions which has led to achieve more specificity and sensitivity of arrhythmia detection compared to previous works [2]- [3]- [4]- [8].Cumulants were utilized in reducing the ECG signal variations in combination with the ability of the Hermitian basis functions to reduce the length of feature vectors and the corresponding computational time. As mentioned in the introduction of the paper we exploited the main properties of HOS to overcome the problem of detecting morphological variations in ECG signal.

The first advantage of this method inherited from cumulants, allowed us to remove many differences in the same type of ECG beats, efficiently and according to the fact that the similarity among the same kind of ECG beats is a very important problem in morphological arrhythmia classification therefore the proposed method achieved to detect and classify the heart arrhythmias much more accurate.

The second important advantage of this method, as the results proved, gives the algorithm a good degree of robustness against the Gaussian type noises existed in ECG signals. As it is shown in Figure 8b, the effect of time shift by 2.8 millisecond is about 0.02% decrease in specificity and 0.08% in sensitivity, which shows that the proposed method has a good stability against time shift. In the situation of amplitude shift, as shown in Figure 8c, shifting up the beat by 1 millivolt results no changes in the beat recognition.

However, one of the limitation of this study is that the scope of comparison the method with others, is limited to the Hermitian and HOS based methods.

According to the features of the Hermitian basis functions, the parameters of the Hermitian model of an ECG beat could be used for compressing it. As it is shown in this paper, an ECG beat with 101 samples could be reconstructed by the 26 coefficients of the Hermitian model and a width parameter, which means that data reduced to about a quarter of the main signal.

As mentioned in "method" section, time consumption of the algorithm was found less than the duration of a beat, which makes the algorithm reasonably fast.

## List of Abbreviations

1-NN: 1-Nearest Neighborhood; APC: Atrial Premature Contraction; AR: AutoRegressive; ECG: ElectroCardioGraphy; EGG: ElectroGastroGram; FN: False Negative; FP: False Positive; HOS: Higher-Order Statistics; k-NN: k-Nearest Neighborhood; LBBB: Left Bundle Branch Block; PVC: Premature Ventricular Contraction; RBBB: Right Bundle Branch Block; Se: Sensitivity; Sp: Specificity; TN: True Negative; TP: True Positive.

## Competing interests

The authors declare that they have no competing interests.

## Authors' contributions

This paper is based on master thesis of SKF under supervision of AA. SKF searched about the theoretical parts, wrote algorithms and converted them in Matlab codes and tested, extracted results and finally made conclusions. AA searched about the theoretical parts, supervised the developing algorithms and writing codes, tests, results and made conclusions. Both authors read and approved the final manuscript.
